# Neural correlates of face processing associated with development of social communication in 12-month infants with familial risk of autism spectrum disorder

**DOI:** 10.1186/s11689-021-09413-x

**Published:** 2022-01-12

**Authors:** Joshua Glauser, Carol L. Wilkinson, Laurel J. Gabard-Durnam, Boin Choi, Helen Tager-Flusberg, Charles A. Nelson

**Affiliations:** 1 Department of Neuroscience, Harvard University, Boston, MA 02138 USA; 2 Division of Developmental Medicine, Boston Children’s Hospital, Harvard Medical School, Boston, MA 02115 USA; 3 Labs of Cognitive Neuroscience, 2 Brookline Place, Brookline, MA 02445 USA; 4 Department of Psychology, Northeastern University, Boston, MA 02120 USA; 5 Department of Psychological and Brain Sciences, Boston University, Boston, MA 02215 USA; 6 Harvard Graduate School of Education, Cambridge, MA 02138 USA

**Keywords:** EEG, Event-related potential, Face processing, Autism, Language development, Infant

## Abstract

**Background:**

Differences in face processing in individuals with ASD is hypothesized to impact the development of social communication skills. This study aimed to characterize the neural correlates of face processing in 12-month-old infants at familial risk of developing ASD by (1) comparing face-sensitive event-related potentials (ERP) (Nc, N290, P400) between high-familial-risk infants who develop ASD (HR-ASD), high-familial-risk infants without ASD (HR-NoASD), and low-familial-risk infants (LR), and (2) evaluating how face-sensitive ERP components are associated with development of social communication skills.

**Methods:**

12-month-old infants participated in a study in which they were presented with alternating images of their mother’s face and the face of a stranger (LR = 45, HR-NoASD = 41, HR-ASD = 24) as EEG data were collected. Parent-reported and laboratory-observed social communication measures were obtained at 12 and 18 months. Group differences in ERP responses were evaluated using ANOVA, and multiple linear regressions were conducted with maternal education and outcome groups as covariates to assess relationships between ERP and behavioral measures.

**Results:**

For each of the ERP components (Nc [negative-central], N290, and P400), the amplitude difference between mother and stranger (Mother-Stranger) trials was not statistically different between the three outcome groups (Nc *p* = 0.72, N290 *p* = 0.88, P400 *p* = 0.91). Marginal effects analyses found that within the LR group, a greater Nc Mother-Stranger response was associated with better expressive language skills on the Mullen Scales of Early Learning, controlling for maternal education and outcome group effects (marginal effects dy/dx = 1.15; *p* < 0.01). No significant associations were observed between the Nc and language or social measures in HR-NoASD or HR-ASD groups. In contrast, specific to the HR-ASD group, amplitude difference between the Mother versus Stranger P400 response was positively associated with expressive (dy/dx = 2.1, *p* < 0.001) and receptive language skills at 12 months (dy/dx = 1.68, *p* < 0.005), and negatively associated with social affect scores on the Autism Diagnostic Observation Schedule (dy/dx = − 1.22, *p* < 0.001) at 18 months.

**Conclusions:**

In 12-month-old infant siblings with subsequent ASD, increased P400 response to Mother over Stranger faces is positively associated with concurrent language and future social skills.

**Supplementary Information:**

The online version contains supplementary material available at 10.1186/s11689-021-09413-x.

## Introduction

Autism spectrum disorder (ASD) is a neurodevelopmental disorder that affects 1 in 54 children in the USA [26]. Symptoms of ASD include deficits in social communication and restrictive/repetitive behaviors that often manifest before the age of three and can persist throughout one’s lifetime [22]. Further, early deficits in social communication can negatively impact the development of social-emotional reciprocity [36], nonverbal communicative behaviors [20], cognitive abilities [40], and language development [6, 20, 38]. Therefore, early detection and implementation of therapies is crucial to mitigating downstream negative effects of early deficits and promoting effective individualized strategies to support development.

Atypical face processing in individuals with ASD is hypothesized to negatively impact social communication [19], and such differences may be present in infancy, prior to the emergence of behavioral symptoms. To identify such differences, researchers have studied infant siblings of children diagnosed with ASD, as they have an increased incidence of a later ASD diagnosis as well as other developmental delays [15, 27, 32, 35]. Several eye tracking studies have observed that high familial risk infants show differences in face scanning as early as 6 months of age (e.g., eyes vs mouth) and that early differences in attention to faces is associated with later social communication ability [12, 41, 46].

Electrophysiological recordings, and more specifically event-related potentials (ERPs), from infant siblings have also been used to identify neural differences in face processing. There are several ERP components that have been shown to be sensitive to face processing: Nc, N290, and P400. The Nc or “negative central” waveform is observed over the frontal regions of the brain and is a marker for attention in both infants and adults [4]. The Nc response is larger in response to novel or unfamiliar objects or faces [5, 25, 31]. Across the first 2 years of life, an infant’s response to their mother versus a stranger’s face shifts, with an increased Nc response to their mother’s face before 1 year of age, but a decreased response to their mother versus a stranger by 2 years of age [2].

The N290, measured over the lateral-inferior posterior scalp, is the most commonly studied face-sensitive ERP component and is thought to be a precursor to the adult N170 waveform that has robustly been observed in response to faces [3, 9]. The role of the P400 in face processing is less clear, as differential responses to faces in studies have not been consistent [17, 18, 21, 28]. Given these discrepancies, it has been hypothesized that the P400 may instead play a role in novelty or saliency processing [3].

Using a subset of data presented in this paper, our team has previously evaluated ERP responses to mother versus a similarly looking stranger in high- and low-familial risk infants at 12 months old [24, 25]. Overall, no significant risk group differences were observed. At 12 months, both risk groups showed a more negative Nc response to strangers, with the differential response trending larger in the low-risk group. While there was a trend toward higher P400 peak amplitude in the high-risk compared to low-risk group, no significant differences were observed in the P400 or N290 component. However, these analyses were performed at the risk group level only, and therefore it is unknown whether there are differences between ASD *outcome* groups—specifically for those high-risk infants who go on to have autism. In addition, understanding how these face-related ERP components are associated with social communication skills at the individual level can shed further light on their role in early development.

The current study aims to further investigate differences in ERP response to mother vs stranger and its association with social communication skills in low- and high-familial risk infants with and without later ASD diagnosis. First, using a larger data set (102 infants vs 56 infants in [25]) we assessed whether Nc, N290, and P400 responses to mother or stranger faces at 12 months differed between three outcome groups—low risk without ASD (LRC), high-familial-risk without ASD (HR-NoASD), and high-familial-risk with ASD (HR-ASD). Second, we assessed whether 12-month ERP responses to mother or stranger faces were associated with (1) early social communication skills as measured on standardized behavioral measures and parent questionnaires at 12 months and (2) later social communication skills as measured by the Autism Diagnostic Observation Schedule (ADOS, [23]) Social Affect score and the Communication and Symbolic Behavior Scales (CSBS) Social Composite score at 18 months.

## Methods

### Participants

Infants were enrolled in a longitudinal study conducted jointly by Boston Children’s Hospital and Boston University and approved by the institutional review board (#X06-08-0374). Written consent was obtained from a parent or guardian prior to each child’s participation.

Exclusion criteria for the study included prenatal or postnatal medical or neurological problems (e.g., seizures), genetic mutations known to affect neurodevelopment, and uncorrected hearing or visual impairment. All infants had a minimum gestational age of 36 weeks and were from households speaking primarily English (English spoken more than 75% of the time). Infants were also excluded from this analysis if they did not complete the 12-month visit or complete the ADOS assessment at a later visit.

Infants were enrolled in two groups: (1) high familial risk infants for ASD who had at least one older sibling with ASD, confirmed using the ADOS or the Social Communication Questionnaire (SCQ) [37], and (2) low risk infants, defined by having a typically developing older sibling, and no first- or second-degree family member with ASD. ASD outcomes groups (LRC, HR-NoASD, and HR-ASD) were determined using behavioral assessments administered at 18–36-month time points (see *Behavioral Assessment*).

Of the 183 eligible infants who provided EEG data for this analysis, only a subset (*n* = 110) met our behavioral and data quality requirements (Supplemental Fig. 1). After ERP preprocessing pipelines described below, 102 ERPs were available for Nc analysis (42 LRC, 40 HR-NoASD, 20 HR-ASD) and 64 ERPs were available for N290/P400 analyses (24 LRC, 26 HR-NoASD, 14 HR-ASD).

### ASD outcome and social communication measures

Final ASD outcome groups were determined using the ADOS [23], administered at 18, 24, and 36 months of age. For participants receiving an ADOS score indicative of ASD or within 3 points of cutoffs, a licensed clinical psychologist reviewed video recordings of concurrent and previous assessments, and using DSM-5 criteria, provided a best estimate clinical judgment in one of three categories: typically developing, ASD, or non-spectrum disorder (e.g., ADHD, anxiety, language delay). Of the 60 HR infants contributing data for this study, 3 children (2 HR-ASD, 1 HR-No ASD) had final outcome judgements based on only the 18 month ADOS assessment. At 18 months, all participants were administered the ADOS Module 1, and the social affect score was used as one measure of social development.

Infants were evaluated using the Mullen Scales of Early Learning (MSEL; [29]) at 6, 9, 12, 18, 24, and 36-month visits. These evaluations assessed receptive and expressive language, fine motor skills, and visual reception developmental domains. This study utilizes standardized *t* scores from expressive language and receptive language subscales of the MSEL at 12 months of age as an early measure of social communication. At 12 months, expected MSEL items are largely building blocks of social communication skills (Receptive—responding to voice and face, attending to words and movement, recognizing own name, understanding gesture and commands; Expressive—smiles, vocalizations, plays gestures/language game). At later ages, items focus on more language based skills (recognizing body parts, following directions with objects, saying words, labeling objects). Given this paper’s focus on social communication, only 12 month MSEL scores were used.

Parents completed the MacArthur-Bates Communicative Development Inventory (MB-CDI): Words and Gestures [14] at the 12-month time point. The study utilizes the Early Gesture and Phrases Understood raw scores from this questionnaire. At the 18-month visit, parents completed the Communication and Symbolic Behavior Scales Developmental Profile (CSBS-DP; [42]). The CSBS-DP is a norm-referenced measure of early social communication and symbolic development. The Social composite standard score (comprised of questions related to emotion, eye gaze, communication, and gestures) was used in subsequent data analyses.

### Mother/stranger stimuli and EEG task procedure

For this task, infants observed color pictures of their mother and a similarly looking stranger. Images of the mother and stranger were randomly presented for 500 ms, maintaining a ratio of 1:1 for each type of picture. Pictures of the mothers were matched with strangers according to ethnicity and whether or not they wore glasses. The mothers and strangers had neutral expressions for their pictures.

EEG sessions were conducted in a sound attenuated and electrically shielded room with minimal lighting. During the sessions, caregivers held the infant on their lap, approximately 65 cm from the experimental monitor. Continuous EEG was recorded using either 64-channel Geodesic Sensor Net System or a 128-channel Hydrocel Geodesic Sensor Nets (Electrical Geodesics, Inc., Eugene, OR, USA). Signals were amplified with a Net Amps 200 or Net Amps 300 amplifier (Electrical Geodesic Inc., Eugene, OR, USA), sampled at either 250 Hz or 500 Hz. EEG data were online-referenced to a single vertex electrode (Cz), and impedances were kept below 100 kΩ. Stimulus presentation was managed via the ePrime software (Psychology Software Tools, Pittsburgh, PA). Each stimulus was initiated only when the child was attending to the screen, as observed by an examiner in the adjacent room. Trials during which the child’s attention was not maintained on the visual stimulus were marked and then removed from further analysis. A maximum of 100 trials (Mother and Stranger combined) were presented. Fewer trials were presented when the infant became fussy, tired, or inattentive. There was no significant difference in number of trials administered between outcome groups (*p* > 0.1, Supplemental Table 1).

### EEG pre-processing

**Fig. 1:**
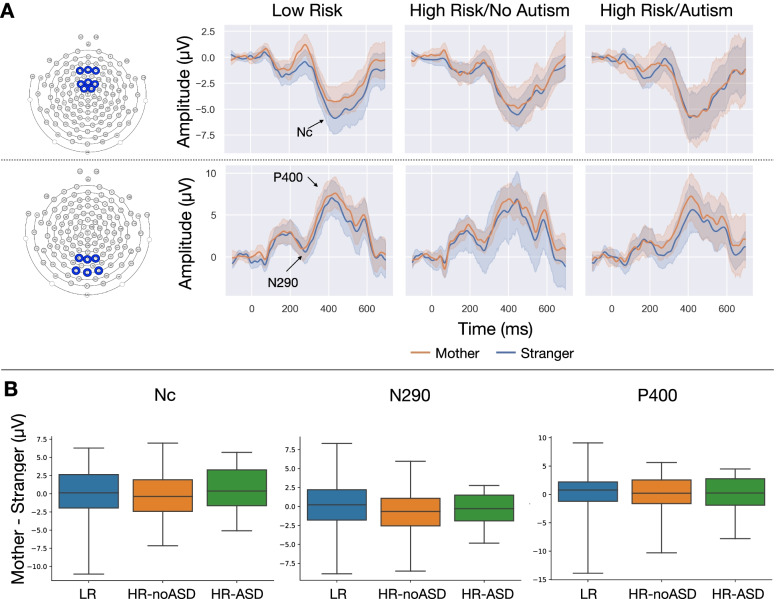
**A** Grand average ERP waveform across group in response to mother versus stranger faces. Electrode groups and ERP response for Nc (top row) and N290/P400 (bottom row). **B** Difference in ERP response to mother versus stranger across outcome groups. No significant differences were observed

The continuous EEG data collected over the mother/stranger paradigm was first downsampled to 250 Hz in Netstation and then exported to MATLAB (versionR2017b) for preprocessing analysis using a modified version of the Harvard Automated Processing Pipeline for EEG (HAPPE; [16]) to allow for ERP analyses similar to the recently released HAPPE+ER software (Monachino et al., under review). Within the modified HAPPE pipeline, artifact within the continuous EEG data is first extracted using the following steps: a copy of the data is made and that copy is high-pass filtered at 1 Hz, channels for subsequent ICA analysis are selected (Supplemental Fig. 2), 60 -Hz electrical noise is removed via Cleanline’s multi-taper regression (Mullen 30), bad channels are rejected, and then remaining artifact is extracted first using wavelet-enhanced independent component analysis (W-ICA), and then subsequently using ICA with MARA automated independent component rejection. Next, the original *unfiltered* EEG file is subjected to the same channel selection and electrical noise removal steps above and the bad channels detected from analysis on the data copy are removed. The artifact signals identified after the W-ICA step on the data copy are then subtracted from the original unfiltered EEG file, and the identified artifact ICA components rejected from the data copy are back-projected to sensor space as timeseries that are then rejected from the original unfiltered signal. This now “clean” unfiltered file is filtered using standard ERP filter settings (0.3–30 Hz), and segmented (− 100 to 700 ms) around the visual stimulus, and baseline corrected via baseline subtraction. Segments with retained artifact in the subset of electrodes used for ERP analyses (Fig. 1A and B) are rejected using HAPPE’s amplitude (amplitude threshold of ± 80 μV) and joint probability criteria, bad channels are interpolated, and data is referenced to the average reference.

#### EEG rejection criteria

Children were excluded from the final sample if they had fewer than 10 trials for either the mother or stranger stimuli or did not meet the following HAPPE data quality output parameters previously determined in this dataset (Wilkinson et al. 43): percent good channels > 82%, percent of independent components rejected < 84%, percent variance of data retained after artifact removal > 32%, mean retained artifact probability < 0.3. There were no significant differences in data quality between outcome groups. Supplemental Table 1 shows quality metrics for all outcome groups for both ERP analyses.

### ERP analysis

Average waveforms for each individual participant for each stimulus condition (mother and stranger) were calculated across electrodes in corresponding regions of interest (Nc: Fig. 1A top, P400: Fig. 1A bottom), which were chosen based on previous literature [17, 18, 24, 25]. To control for the effect of preceding peak/trough amplitude on the Nc, N290, and P400 amplitudes, all peak amplitudes were calculated by measuring the peak-to-peak amplitude [34], which is the magnitude of the component value subtracted from the maximum value of the previous opposite polarity peak (Supplemental Fig. 3).

For the Nc waveform, the peak negative Nc component was identified as the most negative point between 300 and 600ms after the stimulus. The peak negative N290 components were identified as the most negative point between 200 and 350 ms after the stimulus. The peak positive P400 components were identified as the most positive point between 300 and 500 ms.

To evaluate the difference in response for mother against stranger (Mother-Stranger), for each component, the peak amplitude response to stranger was subtracted from the peak amplitude response to mother.

### Statistics

Demographics were analyzed across groups using Fischer’s exact test to determine any differences between groups. Continuous variables (e.g., EEG HAPPE metrics, ERP component amplitudes) were analyzed for normality, and the Kruskal-Wallis *H* tests (one-way nonparametric ANOVA) were used to compare groups when the Shapiro-Wilk test was *p* < 0.05, followed by post hoc Dunn’s tests to examine pairwise comparisons. Bonferroni’s correction was used to account for multiple comparisons such that family-wise error rate was set to *α* < 0.05. Two-way mixed ANOVA were used to determine the effects of group, picture, and group × picture interaction on ERP peak amplitudes.

Simple and multiple linear regressions were used to determine whether ERP peak amplitudes (Mother-Stranger) were associated with social communication measures. To evaluate the effect of outcome group on the relationship between ERP amplitudes and social communication measures, linear regressions models included a two-way interaction between outcome group and the relevant ERP measure. To characterize interaction effects within the models, marginal effects analyses were performed. As maternal education was significantly different between outcome groups, and has been associated with language outcomes in infants, it was included as a covariate in all models.

## Results

### Sample description

Demographic data for each outcome group (LRC, HR-NoASD, and HR-ASD) are shown in Table 1. There was a significant group difference in maternal education, with both the HR-NoASD and HR-ASD having a high proportion of mothers with less than a 4-year college degree. Notably, the majority of participants across groups were white with household incomes above $75,000.

**Table 1:** Sample characteristics

	LR *N* = 45	HR-NoASD *N* = 41	HR-ASD *N* = 24	Fisher’s exact test *P* value
**Sex**	20 M, 25 F	19 M, 22 F	15 M, 9 F	0.326
**Maternal education,***n (%)*				0.047
Not answered	5 (11)	4 (10)	4 (17)	
< 4-year college degree	1 (2)	7 (17)	3 (13)	
4-year college degree	8 (18)	8 (20)	8 (33)	
> 4-year college degree	31 (69)	22 (54)	9 (38)	
**Paternal education,***n (%)*				0.196
Not answered	6 (13)	4 (10)	4 (17)	
< 4-year college degree	3 (7)	7 (17)	4 (17)	
4-year college degree	10 (22)	14 (34)	8 (33)	
> 4-year college degree	26 (58)	16 (39)	8 (33)	
**Household income,***n (%)*				0.81
Not answered	8 (18)	5 (12)	5 (21)	
< $75,000	6 (13)	6 (15)	2 (8)	
> $75,000	31 (69)	30 (73)	17 (71)	
**Race,***n (%)*				0.13
Non-White	6 (13)	3 (7)	6 (25)	

*Abbreviations*: *ASD* Autism spectrum disorder, *LR* Low-risk without ASD, *HR-NoASD* High-risk without ASD, *HR-ASD* High-risk with ASD

### Grand average ERP components across groups

Grand averaged Nc and N290/P400 responses to mother and stranger stimuli by outcome groups are shown in Fig. 1A. The effects of group, stimulus, and group × stimulus interaction on peak-peak amplitude, and latency measures were assessed. No significant main effects or interactions were observed. The distribution of Mother-minus-Stranger peak-peak amplitude (Mother-Stranger) across outcome groups is shown in Fig. 1B.

### ERP Mother-Stranger responses and social communication measures

**Fig. 2:**
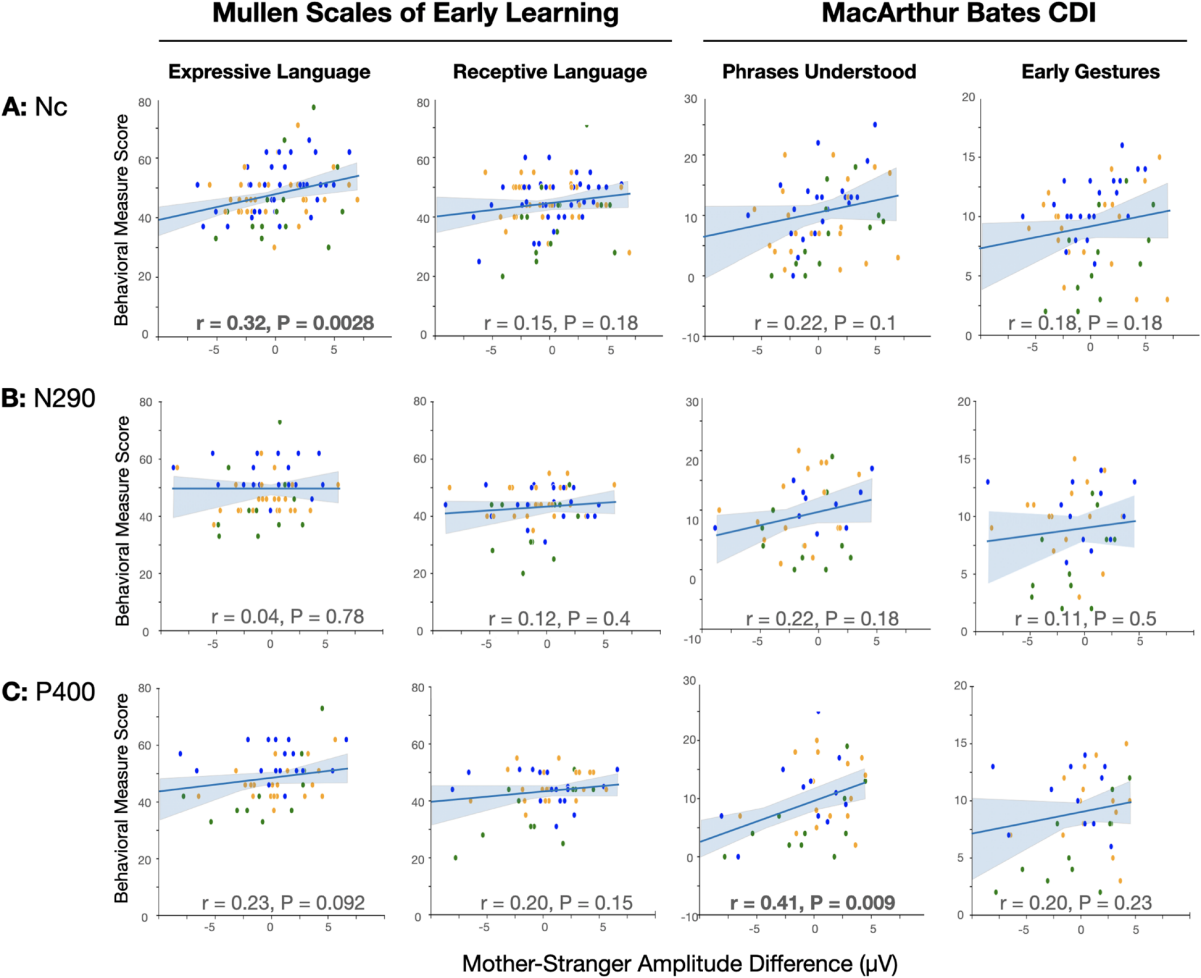
Mother-Stranger amplitude difference and communication measures. Correlations and Pearson’s *r* statistics are shown between ERP amplitudes (**A** Nc, **B** N290, **C** P400) and the following 12-month communication measures: Mullen Scales of Early Learning Expressive Language and Receptive Language *t* scores, MacArther Bates CDI Phrases Understood and Early Gestures raw scores. Blue, LR; orange, HR-NoASD; green, HR-ASD

**Fig. 3:**
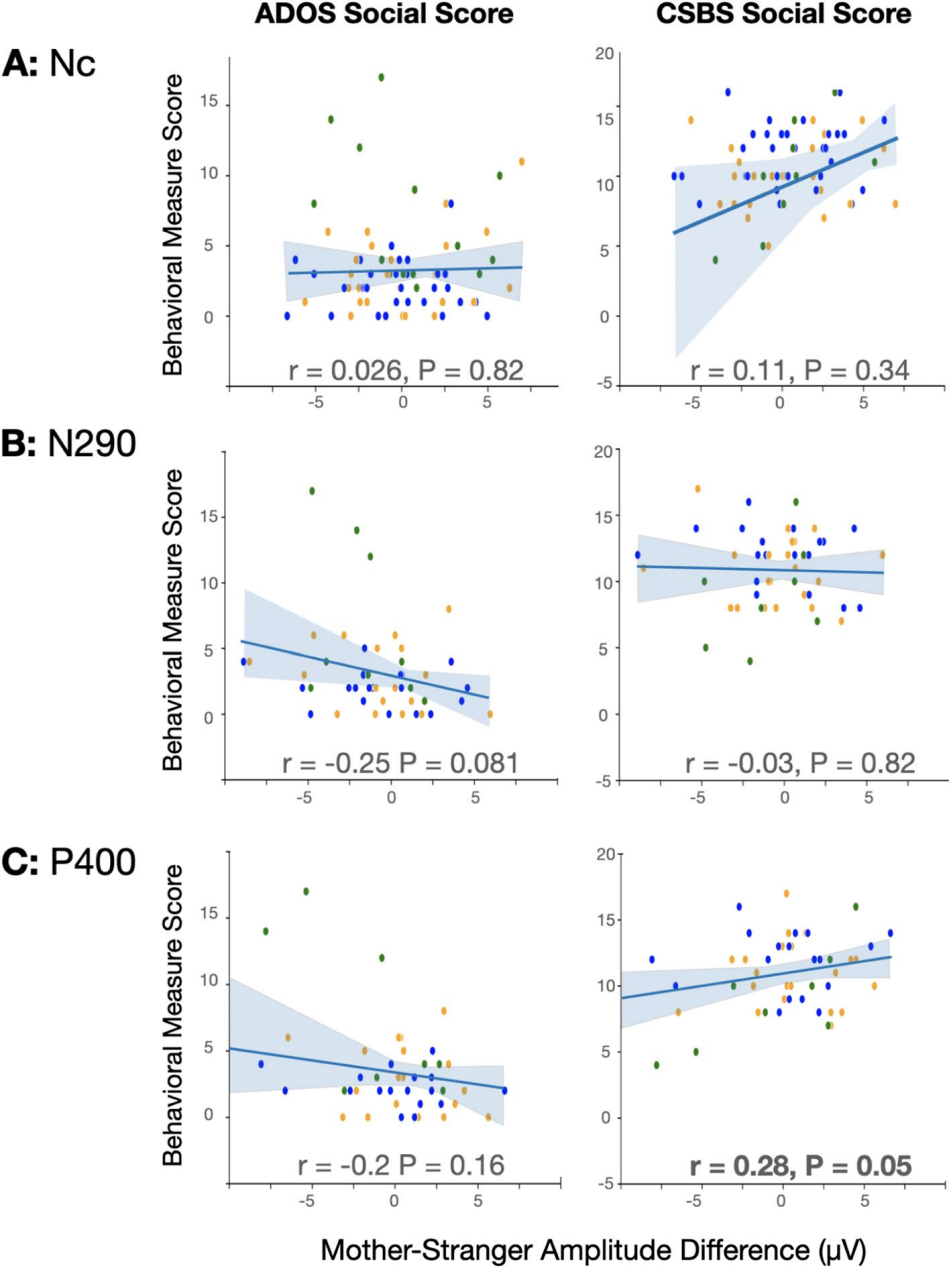
Mother-stranger amplitude difference and 18 month social measures. Correlations and Pearson’s *r* statistics are shown between ERP amplitudes (**A** Nc, **B** N290, **C** P400) and ADOS or CSBS social scores at 18 months. Blue, LR, orange, HR-NoASD, green, HR-ASD

While there were no group differences observed at 12 months of age in N290, P400, and Nc responses to mother/stranger stimuli, there was fairly broad distribution in responses across infants. We investigated whether an infant’s brain response to their mother’s versus a stranger’s face was associated with early and later social communication measures. Here, we define social communication as skills that facilitate social engagement with others (e.g., eye contact, gestures, directed vocalization, response to name). To capture early social communication skills, we used Receptive and Expressive *t* scores on the MSEL, as well as raw scores from the Phrases Understood and Early Gestures sections of the MB-CDI administered at 12 months. At this younger age, both of these measures assess building blocks of social communication (see Methods). To capture later social communication skills, we utilized the social affect score on the ADOS and Social Composite on the CSBS-DP parent questionnaire, both administered at 18 months. Using simple, unadjusted, Pearson correlations across outcome groups, we assessed the relationship between ERP amplitudes and 12-month communication measures (Fig. 2) and 18-month social measures (Fig. 3). We observed that increased Nc response to mother over stranger was positively correlated with Expressive Mullen *T* scores (Pearson’s *r* = 0.32, *p* = 0.0028). Similarly, increased P400 response to mother over stranger was positively correlated with the MB-CDI Phrases Understood (Pearson’s *r* = 0.41, *p* = 0.009). Both correlations remained significant after adjusting for 4 comparisons.

To further evaluate the effect of group on the relationship between Mother-Stranger ERP responses and social communication measures, for each ERP response, two linear regression models were examined. Model 1 included outcome group as an independent variable to account for expected group differences in social communication measures that are independent of ERP responses. Model 2 included two-way interactions between outcome group and the ERP response, with the hypothesis that the relationship between ERP response and social communication measures may be different between outcome groups. For all models, maternal education was also included as a covariate. As expected, significant effects of outcome group on MSEL Expressive and Receptive language scores, MB-CDI measures, and ADOS Social Score were observed (model 1, Table 2). In model 1, after accounting for effects of outcome group and maternal education on social communication measures, the Nc Mother-Stranger response was positively associated with expressive language, and the P400 Mother-Stranger response was positively associated with Number of Phrases Understood (Nc model 1, adjusted *R*^2^ = 0.11; *p* = 0.007; P400 model 1, adjusted *R*^2^ = 0.35; *p* = 0.005).

**Table 2:** Linear regression models of ERP components vs social communication measures

	MSEL Expressive		MSEL Receptive		Phrases Understood		Early Gestures		ADOS Social score		CSBS Social score	
Model	1	2	1	2	1	2	1	2	1	2	1	2
**Nc**												
Adjusted *R*^2^	0.11	0.11	0.09	0.10	0.15	0.15	0.26	0.29	0.24	0.30	− 0.04	− 0.06
Variables [B coefficient (SE)]												
Intercept	50.41 (2.67)	51.56 (2.71)	41.68 (2.74)	42.41 (2.77)	8.60 (2.53)	8.20 (2.78)	8.97 (1.33)	9.26 (1.44)	2.45 (0.98)	2.07 (0.96)	8.93 (4.70)	9.59 (4.85)
Mother-Stranger Response	0.90 (0.29)**	1.15 (0.41)**	0.38 (0.29)	0.63 (0.42)	0.42 (0.23)	0.21 (0.38)	0.22 (0.12)	0.22 (0.20)	− 0.01 (0.11)	− 0.01 (0.17)	0.51 (0.54)	0.83 (0.80)
Maternal Education	− 0.32 (1.38)	− 0.71 (1.41)	2.36 (1.42)	1.92 (1.44)	2.23 (1.35)	2.42 (1.46)	1.20 (0.71)	1.03 (0.77)	− 0.14 (0.50)	0.09 (0.49)	− 0.50 (2.49)	− 0.97 (2.61)
Group												
HR-NoASD	− 2.92 (2.06)	− 3.08 (2.07)	1.07 (2.12)	0.89 (2.11)	− 3.00 (1.74)	− 2.90 (1.74)	− 2.01 (0.91)*	− 1.98 (0.90)*	0.56 (0.77)	0.66 (0.74)	2.16 (3.47)	2.29 (3.52)
HR-ASD	− 5.57 (2.68)**	− 5.89 (2.69)*	− 5.42 (2.75)	− 5.81 (2.75)*	− 4.57 (2.14)*	− 5.19 (2.22)*	− 4.04 (1.13)**	− 4.62 (1.15)**	4.64 (0.98)**	5.02 (0.94)**	1.35 (5.23)	0.95 (5.39)
Interaction												
Nc:HR-NoASD		− **0.80 (0.64)+**		− **0.88 (0.66)+**		0.13 (0.54)		− 0.17 (0.28)		0.28 (0.24)		− 0.77 (1.18)
Nc:HR-ASD		0.26 (0.81)		0.40 (0.82)		**0.94 (0.67)+**		**0.48 (0.35)+**		− **0.56 (0.29)+**		0.18 (1.91)
**N290**												
Adjusted *R*^2^	0.20	0.19	0.16	0.15	0.24	0.24	0.26	0.28	0.21	0.24	0.09	0.14
Variables [B coefficient (SE)]												
Intercept	57.62 (3.12)	58.26 (3.36)	44.57 (2.78)	44.10 (3.003)	3.69 (3.20)	2.28 (3.38)	7.54 (1.75)	7.44 (1.83)	1.01 (1.33)	1.03 (1.40)	7.79 (6.8)	9.79 (1.16)
Mother-Stranger response	0.02 (0.34)	0.01 (0.54)	0.22 (0.30)	− 0.10 (0.49)	0.52 (0.30)	0.29 (0.49)	0.13 (0.17)	− 0.14 (0.26)	− 0.23 (0.15)	− 0.12 (0.22)	− 0.13 (0.76)	− 0.25 (0.18)
Maternal education	− 2.22 (1.59)	− 2.61 (1.72)	− 0.02 (1.42)	0.19 (1.54)	4.34 (1.58)**	5.14 (1.69)**	1.65 (0.86)	1.69 (0.91)	0.51 (0.68)	0.55 (0.72)	− 0.82 (3.5)	1.12 (0.60)
Group												
HR-NoASD	− 7.86 (2.34)**	− 8.16 (2.41)**	0.83 (2.09)	1.03 (2.16)	0.43 (2.18)	1.34 (2.29)	− 0.35 (1.19)	− 0.29 (1.24)	0.90 (1.02)	0.89 (1.03)	4.08 (5.0)	− 0.60 (0.83)
HR-ASD	− 10.21 (2.89)**	− 9.29 (3.03)**	− 7.77 (2.57)**	− 6.86 (2.71)*	− 3.20 (2.43)	− 3.42 (2.49)	− 3.57 (1.33)*	− 3.08 (1.35)*	4.50 (1.30)**	3.15 (1.45)*	2.1 (6.8)	− 1.50 (1.18)
Interaction												
N290:HR-NoASD		− 0.33 (0.80)		0.38 (0.71)		0.76 (0.72)		0.28 (0.39)		− 0.01 (0.33)		0.24 (0.27)
N290:HR-ASD		1.04 (1.04)		1.06 (0.93)		− 0.33 (0.82)		**0.72 (0.44)+**		− **0.91 (0.48)+**		0.89 (0.41)*
**P400**												
Adjusted *R*^2^	0.25	0.36	0.18	0.28	0.35	0.32	0.28	0.36	0.20	0.47	0.17	0.25
Variables [B coefficient (SE)]												
Intercept	57.62 (3.01)	58.37 (2.93)	44.47 (2.74)	44.78 (2.71)	4.95 (2.97)	4.12 (3.33)	7.86 (1.73)	7.16 (1.79)	1.09 (1.33)	0.27 (1.18)	10.31 (1.068)	10.26 (1.08)
Mother-Stranger response	0.48 (0.25)	0.21 (0.34)	0.32 (0.23)	0.01 (0.31)	0.69 (0.23)**	0.39 (0.49)	0.17 (0.13)	− 3.15 (1.27)	− 0.16 (0.12)	− 0.01 (0.13)	0.19 (0.09)*	0.03 (0.12)
Maternal education	− 2.17 (1.53)	− 2.66 (1.52)	0.02 (1.39)	− 0.20 (1.40)	3.93 (1.46)*	4.26 (1.61)*	1.55 (0.85)	1.76 (0.87)	0.49 (0.69)	1.05 (0.61)	0.92 (0.55)	0.91 (0.56)
Group												
HR-NoASD	− 8.00 (2.26)**	− 8.00 (2.09)**	0.67 (2.06)	0.71 (1.9)	− 1.06 (2.00)	− 0.80 (2.08)	− 0.72 (1.16)	− 0.35 (1.12)	0.98 (1.03)	1.00 (0.84)	− 0.85 (0.80)	− 0.80 (0.76)
HR-ASD	− 10.04 (2.78)**	− 9.31 (2.59)**	− 7.79 (2.53)**	− 7.10 (2.39)**	− 4.04 (2.23)	− 3.62 (2.36)	− 3.78 (1.30)**	− 3.15 (1.27)*	4.67 (1.29)**	3.79 (1.08)**	− 2.33 (1.08)*	− 1.95 (1.04)
Interaction												
P400:HR-NoASD		− 0.18 (0.56)		0.05 (0.52)		0.39 (0.63)		0.34 (0.34)		0.14 (0.22)		− 0.15 (0.20)
P400:HR-ASD		1.88 (0.65)**		1.67 (0.60)**		0.40 (0.65)		0.82 (0.35)*		− 1.21 (0.27)**		0.63 (0.24)*

*p* < 0.05 *, *p* < 0.01 **, *p* < 0.25 for Interaction +

**Fig. 4:**
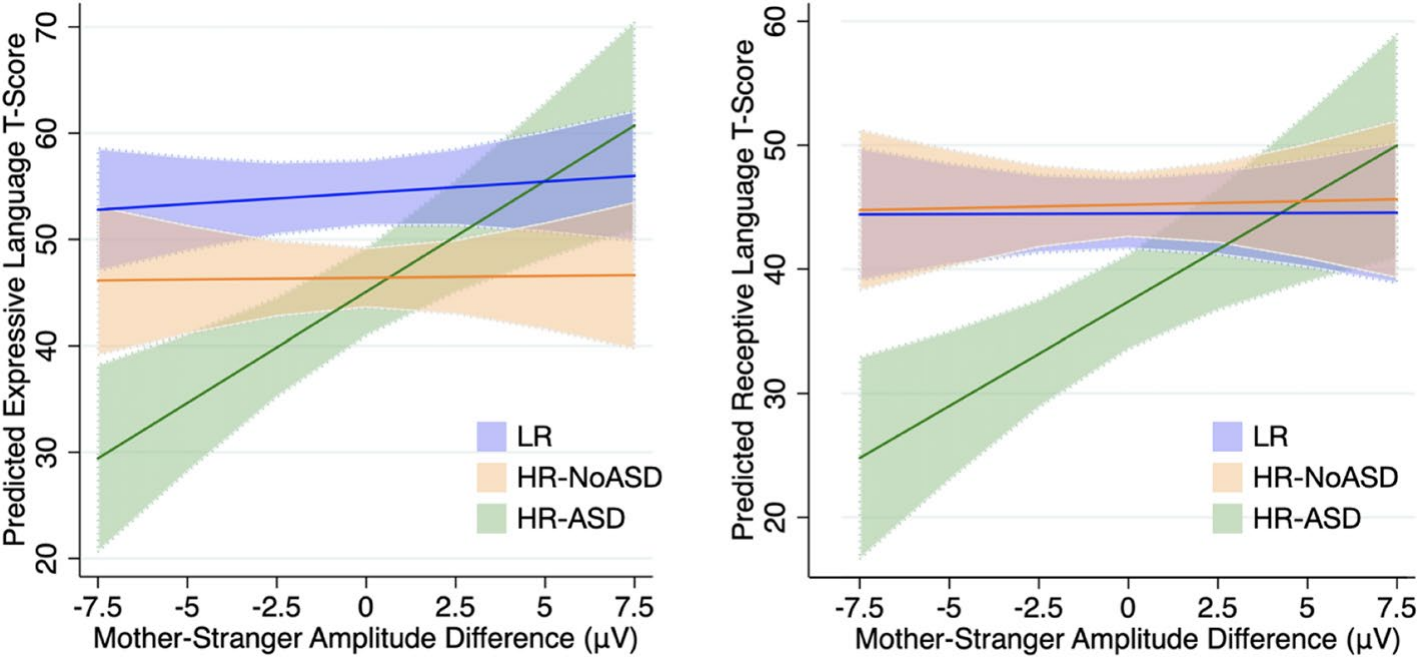
Outcome group differences in predicted language scores based on Mother-Stranger amplitude difference. The relationship between Mother-Stranger amplitude difference and **A** expressive language scores or **B** receptive language scores was significantly different between HR-ASD and both LR and HR-noASD groups (expressive: LR—*p* < 0.01; HR-noASD—*p* < 0.01; receptive: LR—*p* < 0.01; HR-noASD — *p* < 0.05). Blue, LR; orange, HR-NoASD; green, HR-ASD

To assess whether the relationship between ERP response and social communication measures were (1) significant *within* outcome groups or (2) significantly different *between* outcome groups, marginal effects analyses were then performed on model 2 in cases where two-interactions had *p* values < 0.25 (Table 3). In model 2, the significance of the interaction terms represents whether the evaluated association is significantly different between HR-NoASD or HR-ASD groups specifically compared to the LR group. To be inclusive of possible significant associations within outcome groups, that were not significantly different from the LR group, we chose to use a generous *p* value threshold in determining which analyses to perform. Overall, several significant associations, accounting for multiple comparisons, were observed:

1. Slope comparisons of marginal effects from Nc analyses revealed that LR, but not HR-NoASD or HR-ASD infants showed a positive relationship between Nc Mother-Stranger and MSEL Expressive Language *t* scores (slope = 1.15, *p* = 0.007).
2. HR-ASD infants showed a positive association between P400 Mother-Stranger response and both MSEL Expressive and Receptive language *t* scores (slope = 2.10, *p* < 0.001; slope 1.68, *p* = 0.002). Further, these associations for HR-ASD infants were significantly different from both LR and HR-NoASD infants (Fig. 4).
3. For only HR-ASD infants, increased P400 Mother-Stranger response was associated with better social interactions based on lower ADOS Social scores and higher CSBS Social scores. These associations were also significantly different between HR-ASD infants vs either LR (*p* = 0.0001, *p* = 0.01) or HR-NoASD (*p* = 0.001, *p* = 0.04) infants.

**Table 3:** Marginal effects ERP components vs social communication measures

		MSEL Expressive		MSEL Receptive		Phrases Understood		Early Gestures		ADOS Social		CSBS Social	
		δy/δx	*p* value	δy/δx	*p* value	δy/δx	*p* value	δy/δx	*p* value	δy/δx	*p* value	δy/δx	*p* value
Nc	LR	1.15	0.007*	0.63	0.14	0.21	0.59	0.22	0.27	− 0.009	0.96		
	HR-noASD	0.35	0.46	− 0.25	0.61	0.33	0.35	0.05	0.80	0.27	0.12		
	HR-ASD	1.4	**0.04**	1.03	0.15	1.14	0.05	0.70	**0.02**	− 0.57	**0.02**		
N290	LR							− 0.13	0.61	− .12	0.59		
	HR-noASD							0.15	0.27	− 0.12	0.58		
	HR-ASD							0.58	0.11	− 1.03	**0.02**		
P400	LR	0.21	0.54	0.01	0.98			− 0.23	0.40	− 0.01	0.93	0.03	0.78
	HR-noASD	0.03	0.94	0.06	0.88			0.12	0.55	0.12	0.47	0.18	0.23
	HR-ASD	2.10	< 0.001*	1.68	0.002*			0.60	**0.02**	− 1.22	< 0.001*	0.66	0.003*

*Significant after Bonferroni correction for multiple comparisons

## Discussion

Overall, we observed that infants in all three outcome groups had similar ERP responses to pictures of their mother compared to a stranger. The P400 response to mother over stranger was associated with receptive language skills as measured on the MB-CDI. Despite similar ERP responses across groups, we identified outcome group specific relationships between Nc and P400 amplitudes with both communication and social measures. Specifically, for low familial risk infants, Nc was positively associated with expressive language outcomes, whereas, for high familial risk infant with later autism diagnosis, the P400 was positively associated with concurrent expressive and receptive language development and future social skills.

### Lack of differences in ERP amplitudes between cohort groups

Overall, the three groups presented in the study showed similar Nc, N290, and P400 components to the mother/stranger paradigm at 12 months. For these components, there were no differences in the mother, stranger, or Mother-Stranger amplitude values for any of the groups or between groups. Previous studies in infants have similarly found no significant main effects for familial risk group on N290 and P400 amplitudes in response to faces [13, 25, 28], but have observed latency differences between risk groups in response to *objects*. Together, these findings suggest that early face processing is intact in high familial risk infants, including those who later meet ASD criteria. However, studies in preschoolers with ASD have consistently shown differences in ERP responses to familiar/non-familiar faces when compared to typically developing preschoolers [7, 8]. It has been hypothesized that infants with ASD may have delayed development of familiar/unfamiliar ERP responses, which may not be captured at a single 12-month time point. Visually, when examining grand averages, we do observe specifically in the LRC group, a downward shift in the frontally measured ERP response to mother, compared to stranger. While these differences were not statistically different, they do suggest a trend in differential responses, and it was this group where we observed a significant association between Nc response and language skills. While group differences were not identified at this age for this face paradigm, it is possible that other statistical (e.g., machine learning) approaches incorporating *multiple* measures of the ERP response and possible longitudinal measures earlier in development could be predictive of ASD outcome. ASD prediction was not the aim of this analysis, and we note here that predictive analyses will require larger sample sizes to be clinically meaningful.

### Relationships between ERP amplitudes and communication and social measures

Importantly, this study also investigated whether ERP responses were associated with language and social development, and whether such brain-behavior associations were different between outcome groups. Here we uncovered several interesting findings. First, we observed that for low-familial risk infants, a larger Nc to mother over a stranger was positively associated with concurrent expressive language scores on the MSEL. The Nc amplitude has been observed to change from infancy to preschool years, where in infants under 1 year of age, a more negative response is observed in response to familiar faces; On the other hand, by 3-5 years of age, a more negative response is observed in responses to unfamiliar faces [9, 25]. Visually the grand average waveforms for the LRC group (Fig. 1, top), do show a trend toward increased negative response to mothers, perhaps suggesting that a subset of these infants are making this developmental transition sooner than others. Further, our brain-behavior association suggests that early transition of the Nc’s differential familiar/unfamiliar response is associated with more advanced expressive language development. We also note that a similar positive association was observed in the HR-ASD group but was not significant after adjustment for multiple comparisons, likely due to the small sample size within this group. Together, this suggests that Nc response in infancy may not be different between ASD outcomes, but may be an indicator of brain development as it specifically relates to expressive language. Notably, associations were not observed with receptive language and social communication measures. As a form of communication, it is feasible to assume that attentional resources toward a person are more crucial for one’s active communication, being expressive, compared to one’s passive communication, being receptive. However, since gestures are a precursor to expressive language, it is unclear why gestures would not be similarly significant. More research will be needed to explain these discrepancies in infant attentional resources to their communication and social outcomes.

Second, we observed that the P400 component is significantly associated with MB-CDI Phrases Understood while accounting for maternal education and group, indicating a significant association to early receptive language development. In addition, for high-familial risk infants who later met criteria for autism, a greater P400 response to mother over stranger was associated with better concurrent receptive and expressive language, as well as future social skills measured at 18-month. Several studies have investigated clinical correlations of ERP responses to face in infancy. Increased P400 and Nc response to infrequently over frequently shown faces has been associated with better cognitive development [44]. Differential P400 response changes in facial features has been linked to receptive language [21]. While the P400 has been shown to be differentially responsive to faces versus objects in infants as young as 6 months [11], findings have not been consistent [3], and it is unclear if the P400 is a face specific ERP. Both the P400 and Nc components are also hypothesized to be neural markers of sustained attention, as amplitudes are increased in response to novel objects [10, 33, 45], as well as communicative over non-communicative gestures [1]. We hypothesize that a differential P400 response to mother versus stranger represents an infants’ recognition of saliency in their mother’s face and that this is predictive of language and social development.

Interestingly we did not observe any brain-behavior associations with the N290, which is the most frequently studied face-specific ERP component, and thought to be a precursor for the N170 [3, 9]. This may be related to developmental timing, and future analyses will investigate whether relationships change over the first three years of life.

### Limitations

This study contained several limitations. The sample size for the HR-ASD group was small, and while findings within the HR-ASD group were significant, they should be interpreted with caution and will need to be replicated with a larger sample. Additionally, The sample population had substantially higher maternal education than the national average, indicating that the cohort of infants might not be representative of the general population [39]. Furthermore, HR-ASD infants in this particular sample had language development that fell generally in the average range indicating more high functioning individuals in our analysis; therefore, the analysis does not encompass all of the ASD spectrum in terms of language development.

### Conclusions and future directions

We found that there was no difference in the Nc, N290, or P400 responses to mother versus stranger across LRC, HR-NoASD, and HR-ASD groups. However, differential mother vs stranger ERP responses in the Nc and P400 were significantly associated with communication and social development, suggesting they could be a useful biomarker of development for high familial risk infants. Future research will require replication in larger datasets. Further analysis of how differential Nc and P400 responses develop over infancy to preschool age across low and high-risk groups will also provide valuable information on differences in brain development as they relate to language and social development.

## Supplementary Information

**Additional file 1: Supplemental Figure 1.** Consort diagram of infants that were included for ERP analyses. EEGs were removed from analysis prior to HAPPE processing (i.e. technical error in EEG collection, infant fell asleep, got fussy, or wouldn’t look at the screen). After HAPPE processing, EEGs were then excluded if they had fewer than 10 trials for either the mother or stranger stimuli, or did not meet the following HAPPE data quality output parameters: percent good channels > 82%, percent of independent components rejected < 84%, percent variance of data retained after artifact removal > 32%, mean retained artifact probability > 0.3. **Supplemental Figure 2.** Electrode layouts for both 128 (left) and 64 (right) channel nets. To optimize artifact performance given the lengths and sampling rates of the EEG data, spatially distributed subsets of channels included ROIs for both Nc and P400 ERPs were processed through HAPPE. **Supplemental Figure 3.** Component Amplitude Calculation that were utilized in each of the Nc, N290, and P400 analyses. For analyses of these components, the amplitude of the mother was subtracted from the amplitude of the stranger to evaluate the difference in response for mother against stranger (Mother-Stranger). This method of waveform analysis has been previously performed when evaluating the Nc, N290, and P400 components [44].

**Additional file 2: Supplemental Table 1.**

## Data Availability

The datasets used and/or analyzed during the current study are available from the corresponding authors on reasonable request.

## Abbreviations

ADOS: Autism Diagnostic Observation Schedule
ANOVA: Analysis of variance
ASD: Autism spectrum disorder
CDI: MacArthur Bates Communicative Development Inventories
CSBS-DP: Communication and Symbolic Behavior Scales Developmental Profile
EEG: Electroencephalogram
ERP: Event-related potential
HAPPE: Harvard Automated Processing Pipeline for Electroencephalography
HR-ASD: High risk with autism spectrum disorder
HR-NoASD: High risk with no autism spectrum disorder
ICA: Independent component analysis
LRC: Low-risk controls
MARA: Multiple Artifact Rejection Algorithm
MSEL: Mullen Scales of Early Learning
SCQ: Social Communication Questionnaire
W-ICA: Wavelet-enhanced independent component analysis
