# Supplementary material for: Neural correlates of face processing associated with development of social communication in 12-month infants with familial risk of autism spectrum disorder
Source: J Neurodev Disord. 2022 Jan 12;14:6. doi: 10.1186/s11689-021-09413-x (PMC8903527; doi:10.1186/s11689-021-09413-x)
Supplement: Supplementary file 1 — Additional file 1: Supplemental Figure 1. Consort diagram of infants that were included for ERP analyses. EEGs were removed from analysis prior to HAPPE processing (i.e. technical error in EEG collection, infant fell asleep, got fussy, or wouldn’t look at the screen). After HAPPE processing, EEGs were then excluded if they had fewer than 10 trials for either the mother or stranger stimuli, or did not meet the following HAPPE data quality output parameters: percent good channels > 82%, percent of independent components rejected < 84%, percent variance of data retained after artifact removal > 32%, mean retained artifact probability > 0.3. Supplemental Figure 2. Electrode layouts for both 128 (left) and 64 (right) channel nets. To optimize artifact performance given the lengths and sampling rates of the EEG data, spatially distributed subsets of channels included ROIs for both Nc and P400 ERPs were processed through HAPPE. Supplemental Figure 3. Component Amplitude Calculation that were utilized in each of the Nc, N290, and P400 analyses. For analyses of these components, the amplitude of the mother was subtracted from the amplitude of the stranger to evaluate the difference in response for mother against stranger (Mother-Stranger). This method of waveform analysis has been previously performed when evaluating the Nc, N290, and P400 components [44]. [file 11689_2021_9413_MOESM1_ESM.pdf]

Supplemental Figure 1

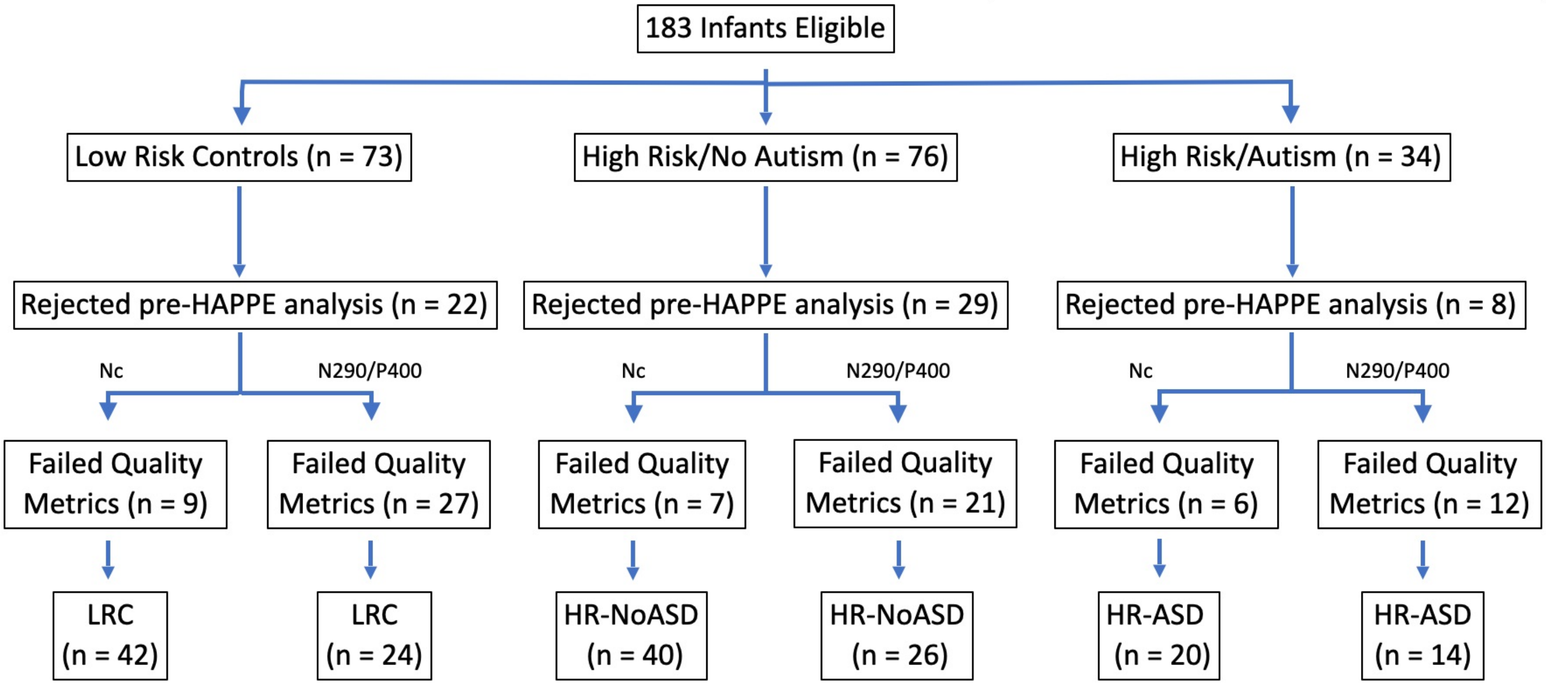

Consort diagram of infants that were included for ERP analyses. EEGs were removed from analysis prior to HAPPE processing (i.e. technical error in EEG collection, infant fell asleep, got fussy, or wouldn't look at the screen). After HAPPE processing, EEGs were then excluded if they had fewer than 10 trials for either the mother or stranger stimuli, or did not meet the following HAPPE data quality output parameters: percent good channels > 82%, percent of independent components rejected < 84%, percent variance of data retained after artifact removal > 32%, mean retained artifact probability > 0.3.

Supplemental Figure 2

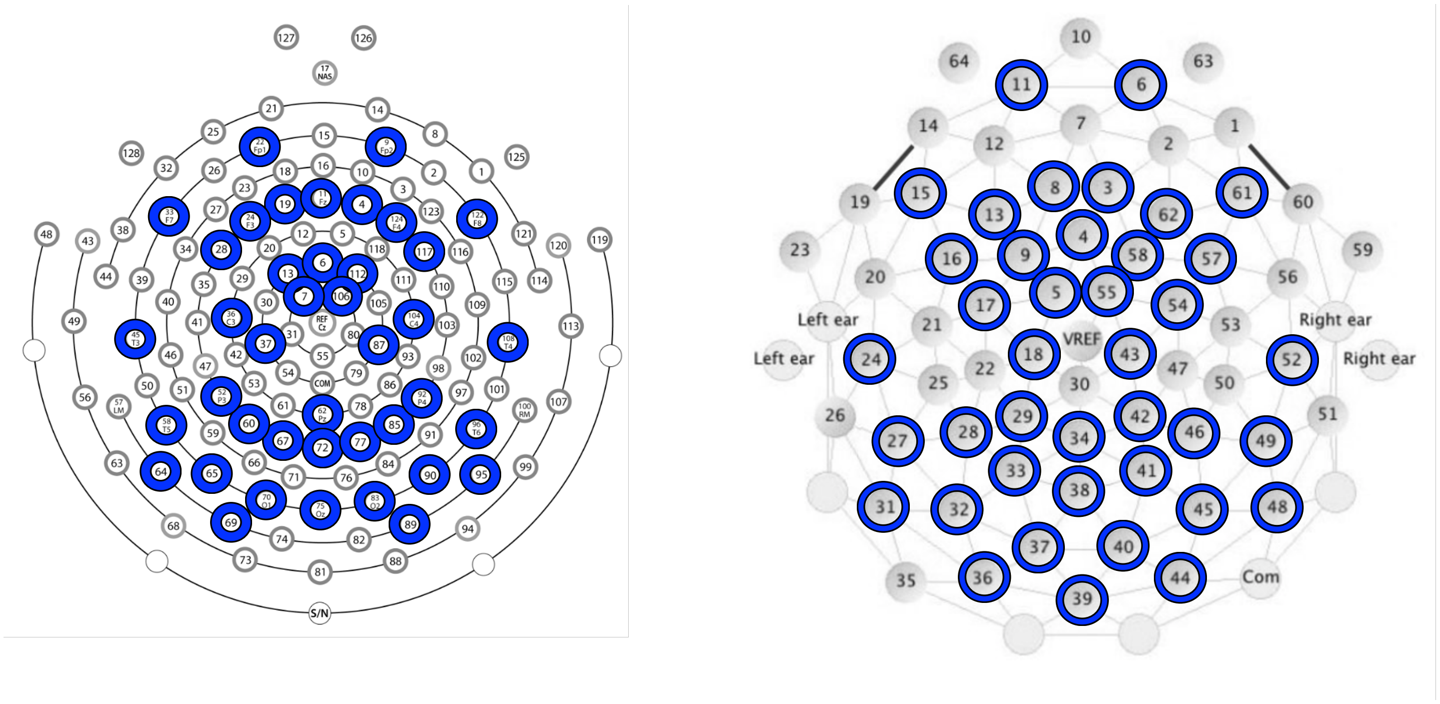

Electrode layouts for both 128 (left) and 64 (right) channel nets. To optimize artifact performance given the lengths and sampling rates of the EEG data, spatially distributed subsets of channels included ROIs for both Nc and P400 ERPs were processed through HAPPE.

Supplemental Figure 3

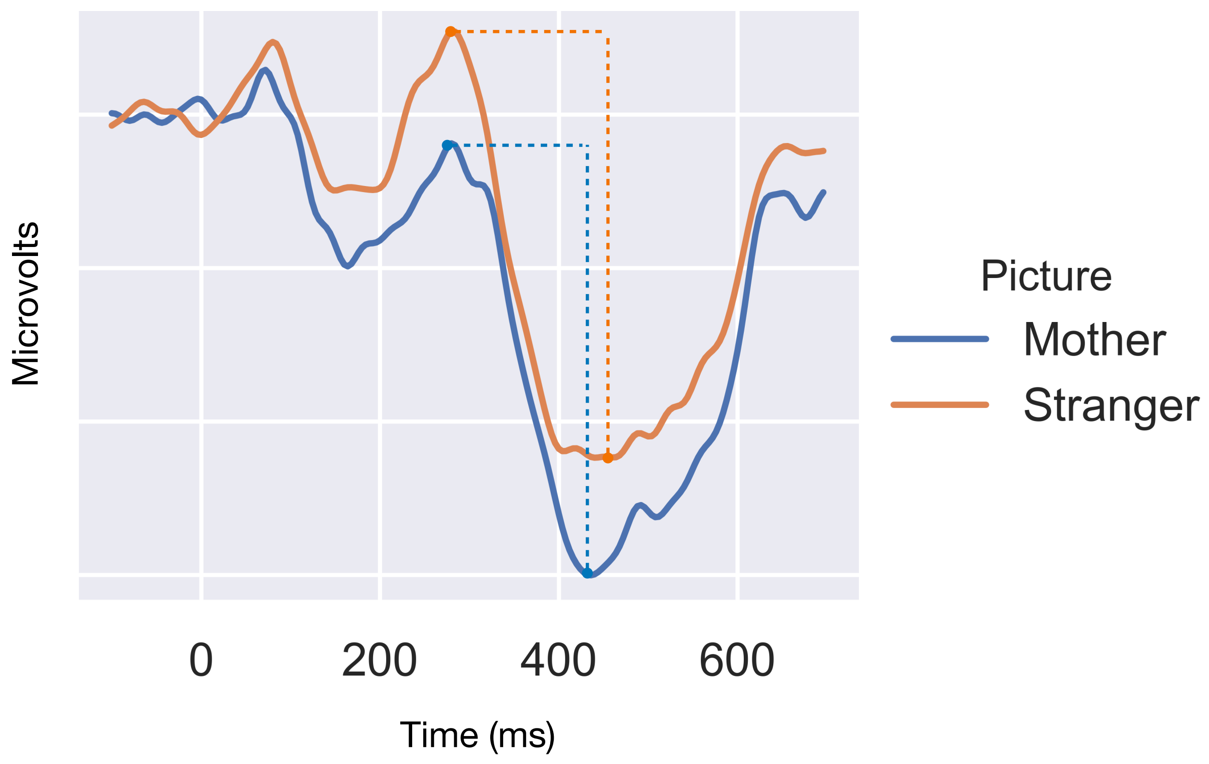

Component Amplitude Calculation that were utilized in each of the Nc, N290, and P400 analyses. For analyses of these components, the amplitude of the mother was subtracted from the amplitude of the stranger to evaluate the difference in response for mother against stranger (Mother-Stranger). This method of waveform analysis has been previously performed when evaluating the Nc, N290, and P400 components (Xie et al., 2019).
