# Supplementary material for: Neural correlates of face processing associated with development of social communication in 12-month infants with familial risk of autism spectrum disorder
Source: J Neurodev Disord. 2022 Jan 12;14:6. doi: 10.1186/s11689-021-09413-x (PMC8903527; doi:10.1186/s11689-021-09413-x)
Supplement: Supplementary file 2 — Additional file 2: Supplemental Table 1. [file 11689_2021_9413_MOESM2_ESM.docx]

**Supplemental Table 1**

| **Nc Analyses** | Total  N = 102 | LRC  N = 42 | HR-NoASD  N = 40 | HR-ASD  N = 20 | Kruskal-Wallis P-value |
| --- | --- | --- | --- | --- | --- |
| **Number of Trials (Mother)** Mean±SD | 24.3 ± 8.2 | 23.2 ± 7.8 | 23.7 ± 8.5 | 27.8 ± 8.0 | 0.14 |
| **Number of Trials (Stranger)** Mean±SD | 24.3 ± 8.3 | 23.2 ± 8.1 | 24.0 ± 8.0 | 27.5 ± 8.7 | 0.17 |
| **Percent of Good Channels Selected** Mean±SD | 94.7 ± 4.6 | 94.6 ± 4.2 | 94.3 ± 5.2 | 95.5 ± 4.3 | 0.62 |
| **Percent of ICs Rejected** Mean±SD | 50.2 ± 14.2 | 51.2 ± 13.0 | 50.9 ± 14.3 | 47.9 ± 16.6 | 0.77 |
| **Percent Variance Kept of Post Waveleted Data** Mean±SD | 61.9 ± 14.5 | 59.5 ± 13.9 | 62.6 ± 14.0 | 65.6 ± 16.7 | 0.25 |
| **Mean Artifact Probability of Kept ICs** Mean±SD | 0.18 ± 0.04 | 0.19 ± 0.04 | 0.19 ± 0.04 | 0.18 ± 0.04 | 0.89 |
|  | | | | | |
| **N290/P400 Analyses** | Total  N = 64 | LRC  N = 24 | HR-NoASD  N = 26 | HR-ASD  N = 14 | Kruskal-Wallis P-value |
| **Number of Trials (Mother)** Mean±SD | 22.4 ± 7.3 | 20.8 ± 6.6 | 22.5 ± 7.5 | 24.8 ± 7.8 | 0.31 |
| **Number of Trials (Stranger)** Mean±SD | 22.0 ± 6.4 | 21.0 ± 6.2 | 22.3 ± 6.3 | 23.1 ± 7.0 | 0.70 |
| **Percent of Good Channels Selected** Mean±SD | 94.1 ± 5.0 | 94.2 ± 5.3 | 94.1 ± 5.0 | 94.1 ± 4.8 | 0.97 |
| **Percent of ICs Rejected** Mean±SD | 53.0 ± 16.8 | 54.1 ± 14.9 | 49.6 ± 15.2 | 57.1 ± 22.1 | 0.29 |
| **Percent Variance Kept of Post Waveleted Data** Mean±SD | 59.6 ± 17.7 | 56.6 ± 17.7 | 63.7 ± 13.5 | 57.1 ± 23.3 | 0.40 |
| **Mean Artifact Probability of Kept ICs** Mean±SD | 0.19 ± 0.05 | 0.19 ± 0.05 | 0.18 ± 0.05 | 0.20 ± 0.05 | 0.50 |
